# LINC02190 inhibits the embryo–endometrial attachment by decreasing ITGAD expression

**DOI:** 10.1530/REP-21-0300

**Published:** 2022-01-14

**Authors:** Feiyan Zhao, Tong Chen, Xuehan Zhao, Qin Wang, Yonglian Lan, Yu Liang, Ying Li, Shuyu Wang, Yang Yang, Xiaokui Yang

**Affiliations:** 1 Department of Human Reproductive Medicine, Beijing Obstetrics and Gynecology Hospital, Capital Medical University, Beijing, China; 2 Department of Human Reproductive Medicine, Beijing Maternal and Child Health Care Hospital, Beijing, China

## Abstract

Recurrent implantation failure (RIF) is a challenge in the field of reproductive medicine, but mechanisms for its occurrence remain still unclear. Long non-coding RNAs (lncRNAs) have been found to play a vital role in many different diseases. In recent years, the differentially expressed lncRNAs have been reported in endometrial tissues. Here, we profiled dysregulated lncRNAs and mRNAs in the endometrial tissues of RIF patients and performed correlation analysis. We found that LINC02190 was upregulated in RIF endometrium and was bound to the integrin αD (ITGAD) mRNA promoter. Immunofluorescence assays were used to detect the location of ITGAD in the Ishikawa cell line and patients’ endometrial biopsies. Overexpressed LINC02190 could decrease the expression of ITGAD and the adhesion rate of Ishikawa and JAR cells. Knockdown of the expression of LINC02190 significantly increased the ITGAD level, as well as the adhesion rate of Ishikawa and JAR cells. Furthermore, we demonstrated that the 150–250 bps of LINC02190 were the cis-elements involved in the regulation of ITGAD promoter activities. In conclusion, the results demonstrated that LINC02190 plays an important role in the occurrence of RIF, and the molecular mechanism may be associated with the embryo–endometrial attachment mediated by ITGAD. This study emphasizes the importance of lncRNAs in the occurrence of RIF and provides a potential new biomarker for diagnosis and therapies.

## Introduction

With the rapid development of assisted reproductive technologies (ART), *in vitro* fertilization/intracytoplasmic sperm injection-embryo transfer (IVF/ICSI-ET) has been widely applied to infertile couples. Although ART has improved the pregnancy outcome of infertile females to a certain extent, females with recurrent implantation failure (RIF) still struggle to achieve a favorable clinical pregnancy after multiple embryo transfers.

Without accepted formal definition at present, Polanski suggested that RIF could be defined as a failure to achieve embryo implantation after two or more consecutive cycles of IVF/ICSI-ET or frozen-thawed embryo transfer (FET) cycles, where the cumulative number of transferred embryos was no less than four high-quality cleavage-stage embryos or no less than two for blastocysts (Polanski *et al.* 2014).

The reported incidence of RIF is about 10% (Diedrich *et al.* 2007). Endometrial receptivity, embryo quality, and the synchronous development of the embryo and endometrium are three essential factors associated with RIF (Koot *et al.* 2012). Recent studies have found an imbalance in the proportion of T helper cells (Liang *et al.* 2015), abnormal expression of cytokines (Thouas *et al.* 2015, Ledee *et al.* 2016), integrins (Coughlan *et al.* 2013), miRNA (Rekker *et al.* 2018), and long ncRNAs (lncRNAs) (Fan *et al.* 2017, Feng *et al.* 2018) in endometrium or serum are all associated with RIF incidence.

As a newly focused topic of research, lncRNAs have attracted more and more attention recently. LncRNAs are RNAs longer than 200 nucleotides and do not directly translate into proteins (Spizzo *et al.* 2012). Many studies indicated that lncRNAs played a vital role in dozens of biological processes through multiple mechanisms such as chromatin remodeling, genetic imprinting, splicing regulation, mRNA editing, mRNA degradation, and translational regulation (Geisler & Coller 2013, Zhu *et al.* 2013). In recent years, there have been increasing numbers of studies investigating the function of lncRNA in RIF. Zeng *et al*. showed that lncRNA H19 regulated integrin β3 (ITGB3) through its antagonistic effect on let-7, which might affect endometrial receptivity and embryo implantation (Zeng *et al.* 2017). Another study showed that lncRNAs (BCAR4, C3orf56, TUNAR, OOEP-AS1, CASC18, and LINC01118) were significantly expressed in metaphase II (MII) oocyte and they could affect embryo development and implantation by participating in biological processes such as chromatin remodeling, cell pluripotency, and in driving early embryonic development (Bouckenheimer *et al.* 2018).

Integrins, a family of α/β heterodimeric cell-surface adhesion receptors, are expressed in several types of cells (Barczyk *et al.* 2010). The integrin family plays an important role in mediating cell–cell and cell–matrix interactions, and is considered to be necessary for cell migration and invasion (Kuo *et al.* 2020). It has been reported that the integrin family was linked to several pathophysiologic processes, such as cancer (Hamidi & Ivaska 2018), cardiovascular disease (Clemetson & Clemetson 1998), inflammatory disease (Mitroulis *et al.* 2015), embryonic development, and angiogenesis (Tiwari *et al.* 2011). It can also affect early pregnancy by mediating invasion, attachment, cell–cell interactions, and decidualization (Rarani *et al.* 2018), which may be one of the underlying mechanisms of RIF.

The present study determined a total of 291 unique differentially expressed lncRNAs in RIF endometrium. Among these, 178 lncRNAs were significantly upregulated and the remaining 113 lncRNAs were downregulated. We elucidated that LINC02190 could decrease ITGAD expression by binding to the 150–250 bp region of the promoter and inhibit the embryo–endometrial attachment, which may result in the occurrence of RIF.

## Materials and methods

### Sample collection

This study included patients receiving IVF-ET treatment in the Department of Human Reproductive Medicine, Beijing Obstetrics and Gynecology Hospital, Capital Medical University during the period from January 1, 2018, to December 31, 2018. A total of 30 patients were recruited. This study was approved by the ethics committee of Beijing Obstetrics and Gynecology Hospital, Capital Medical University. Fifteen patients diagnosed as RIF and 15 normal fertile women were included in RIF and control group, respectively. All participants were informed about the purpose of the study and the research procedures, gave their written informed, and signed their informed consent in the study.

Inclusion criteria were age <40 years, regular menstrual cycle (21–35 days), infertility due to tubal factor or male factor. Patients diagnosed with abnormal morphology of uterine cavity, hysteromyoma, adenomyosis, endometrial polyps, intrauterine adhesion, endometriosis, polycystic ovarian syndrome, or hydrosalpinx were excluded from the study. Endometrial biopsies were obtained using Pipelle suction curettes (Pipelle de Cornier, Laboratoire C.C.D, Paris, France) at luteinizing hormone (LH) +7 day. PBS was flushed through the endometrial tissue to remove any residual mucus and blood. After that, endometrial biopsies were snap-frozen in liquid nitrogen and transferred to a −80°C refrigerator for long-term storage. Five samples of each group were randomly selected for LncRNA Microarray analysis and quantitative real-time PCR (qRT-PCR) was used to validate the other ten samples.

### RNA isolation and quality assessment

Total RNA extraction from the endometrial tissue using TRIzol reagent (Takara) was performed as described previously (Klohonatz *et al.* 2019). In brief, the frozen tissues were homogenized in TRIzol reagent, chloroform was added to the homogenized samples, mixed well, and left for 8 min at room temperature (RT). Samples were centrifuged at 16,100 ***g*** for 15 min at 4°C. The upper aqueous phase, containing the isolated RNA was collected and purified. Samples were treated with the DNase (Thermo Fisher Scientific) to remove the residual DNA. RNA quantity and quality were measured using the NanoDrop ND-1000 spectrophotometer (NanoDrop Technologies, Inc., Wilmington, DE, USA), and denaturing agarose gel electrophoresis was used to confirm RNA integrity and contamination.

### RNA labeling and array hybridization

Arraystar Human LncRNA Microarray V5.0 was used to detect differential expression of RNA. Sample labeling and array hybridization were performed according to the Agilent One-Color Microarray-Based Gene Expression Analysis protocol (Agilent Technology) with minor modifications. Briefly, mRNA was purified from total RNA after removal of rRNA (mRNA-ONLY™ Eukaryotic mRNA Isolation Kit, Epicentre). Then, each sample was amplified and transcribed into fluorescent cRNA along the entire length of the transcripts without 3’ bias utilizing a random priming method (Arraystar Flash RNA Labeling Kit, Arraystar). The labeled cRNAs were purified by RNeasy Mini Kit (Qiagen). The concentration and specific activity of the labeled cRNAs (pmol Cy3/μg cRNA) were measured by NanoDrop ND-1000. 1 μg of each labeled cRNA was fragmented by adding 5 μL 10 × blocking agent and 1 μL of 25 × fragmentation buffer, then the mixture was heated at 60°C for 30 min, finally 25 μL 2 × GE hybridization buffer was added to dilute the labeled cRNA. 50 μL of hybridization solution was dispensed into the gasket slide and assembled to the LncRNA expression microarray slide. The slides were incubated for 17 h at 65°C in an Agilent Hybridization Oven. The hybridized arrays were washed, fixed, and scanned using the Agilent DNA Microarray Scanner (part number G2505C).

### Bioinformatics analysis

Gene Ontology (GO) terms of biological process and molecular function were used to determine the function of target genes. Kyoto Encyclopedia of Genes and Genomes (KEGG) pathway analysis was applied to explore the significant pathways of differentially expressed genes (DEGs). We performed GO classification and KEGG pathway analysis using Cytoscape V3.5.1 with the ClueGo V2.3.5 plug-in. The *P*-value was calculated by a two-sided hypergeometric test and Benjamini–Hochberg adjustment. GO terms and KEGG pathways were identified as enriched in target genes when *P* ≤ 0.05.

### Cell culture and transfection

The Ishikawa human endometrial adenocarcinoma cell line and the JAR human choriocarcinoma cell line (ATCC, Rockville, MD, USA) were cultured in RPMI 1640 medium (GIBCO, Invitrogen Corporation) containing 10% FBS (Hyclone, Logan, UT, USA) and 1% penicillin/streptomycin (Sigma), and maintained at 37°C under 5% CO_2_ in the air. Cells were first seeded into 24-well plates. When the cell density reached 60–70%, Ishikawa cell was transfected with pcDNA3.1 and pcDNA3.1-LINC02190 in strict accordance with the instructions of Lipofectamine™ 2000 (Invitrogen). 6 h after transfection, the medium was replaced and the cells were collected for subsequent experiments.

### Quantitative real-time PCR

qRT-PCR analysis was used 7500 Fast Real-Time PCR System (Applied Biosystems) with a TB Green™ Premix Ex Taq™II kit (Takara, RR820A), according to the manufacturer’s protocol. The primer sequences for qRT-PCR are listed in Table 1. The following parameters were used: 95°C for 30 sec; 40 cycles of 95°C for 5 sec, and 60°C for 34 sec. All the experiments were repeated in triplicate. The housekeeping gene glyceraldehyde 3-phosphate dehydrogenase (GAPDH) was used as an endogenous reference. The relative mRNA expression levels were calculated using the 2^−ΔΔCt^ method.

**Table 1:** Primers used in quantitative real-time PCR.

Primers	Sequence (5’→3’)	
LINC02190 (1–450 bp)	Forward	CTGCTGATCCCAATTTCCTAT
	Reverse	TCAACACCTCTTTGTCAATCTC
LINC02190 (250–568 bp)	Forward	CCTCCCTATGTGTGTTCGCAC
	Reverse	CGAGTCCAAAGGCATGCATG
LINC02190 (1–350 bp)	Forward	CTGCTGATCCCAATTTCCTAT
	Reverse	TGATGGGTCACTGGGTTTCT
LINC02190 (150–568)	Forward	GGCCATGTTCAGGAAGTACTC
	Reverse	ACAATCCAAAGAACCCTGTCC
LINC02190 (1–568 bp)	Forward	CTGCTGATCCCAATTTCCTAT
	Reverse	AATGCGGGACATTATCTGACA
ITGAD	Forward	TGTCAGGAGCCCAGAAG
	Reverse	CCTCATAAGCATCCTGTCTC
GAPDH	Forward	GGTCTCCTCTGACTTCAACA
	Reverse	GTGAGGGTCTCTCTCTTCCT

### Western blot

Radio-immunoprecipitation assay lysate (Beyotime, Shanghai, China) was employed to extract the total protein of endometrial tissues and transfected cells. After centrifugation, the supernatant was collected and the protein concentration was determined by the bicinchoninic acid method (Pierce). Extracted proteins were separated by SDS-PAGE and transferred onto PVDF membranes (Millipore). After blocking with 5% skim milk for 1 h, the membranes were incubated with corresponding primary antibodies at 4°C overnight. After washing three times with TBS with Tween 20 (TBST; Beyotime, Shanghai, China), the membranes were incubated with corresponding secondary antibody at RT for 2 h. Immunoreactive bands were exposed by the ECL method (Thermo Fisher Scientific). GAPDH was used as an internal reference.

The antibodies used for WB are listed below: Anti-ITGAD (abx101638, 1:1000, Abbexa), Anti-GAPDH (ab8245, 1:1000, Abcam). Secondary antibodies: Goat Anti-Rabbit IgG (H+L) HRP (ab6721, 1:10,000, Abcam), Rabbit Anti-Mouse IgG (H+L) HRP (ab6728, 1:10,000, Abcam).

### Luciferase reporter assay

Ishikawa cells were cultured at a density of 2 × 10^4^ cells/well in 96-well culture plates and transfected with 0.2 μg of dual-luciferase reporter construct pGL3-ITGAD, or co-transfected with 0.2 μg of the luciferase reporter construct pcDNA3.1-LINC02190-201 and the internal control vector pRL-TK, pcDNA3.1, or pGL3-basic (Promega) at a ratio of 20:1 (reporter construct: control vector) using Lipofectamine™ 2000 (Invitrogen) according to the instruction of the manufacturer. 5 h post-transfection, the transfection medium was removed and replenished with a fresh medium. 48 h post-transfection, luciferase activity was measured using the Dual-Luciferase® Reporter Assay System (Promega). Renilla luciferase activity was normalized to firefly luciferase activity in cells transfected with the dual-luciferase reporter construct pGL3-ITGAD, and firefly luciferase activity was normalized to renilla luciferase activity in cells co-transfected with the reporter construct pcDNA3.1-LINC02190-201 and the control vector.

### Immunofluorescence

Slides of Ishikawa cells were fixed in 4% paraformaldehyde for 30 min at RT and then permeabilized with 0.2% Triton X-100 for 20 min. After washing with PBS, the cells were blocked with 2% BSA for 30 min and then incubated with anti-ITGAD (abx101638, 1:100, Abbexa) for 30 min at RT. After washing, the primary antibodies were detected by incubating with the corresponding secondary antibodies (A10042, 1:100, Invitrogen) for 1 h in the dark at RT, while the nuclei were counterstained with DAPI (1:1000; Sigma-Aldrich) for 10 min at RT. Finally, the slides were washed with PBS and mounted with an anti-fade medium (Beyotime, Shanghai, China).

Paraffin-embedded sections were deparaffinized, rehydrated, and boiled in citrate buffer for antigen retrieval. After permeabilizing in 0.5% Triton-X-100 for 10 min, any non-specific signal was blocked with 3% BSA for 1 h at RT. The sections were then incubated with primary antibodies anti-ITGAD (abx101638, 1:200, Abbexa) and anti-E-Cadherin (#14472, 1:100, CST) overnight at 4°C. Rabbit MAb IgG (ab172730, 1:200, Abcam) was used as a negative control to ensure antibody specificity. After PBS washes, they were incubated with appropriate secondary antibodies (A10042, 1:100, Invitrogen; A21202, 1:100, Invitrogen) and DAPI for 1 h at RT.

Fluorescent signals were observed and imaged with a confocal laser scanning microscope (Zeiss LSM 510 confocal microscope).

### JAR–Ishikawa cell line co-culture

In our study, JAR cells were stained with Calcein-AM to monitor/quantify the attachment of JAR cells to the Ishikawa monolayers. Calcein-AM is hydrolyzed by cytosolic esterases into Calcein after it crossed the cell membrane passively, which could emit green fluorescence and be retained by cells with intact membranes. Calcein-AM is one of the most ideal fluorescent probes for staining living cells as it has low cytotoxicity and is low pH-sensitive.

The brief protocol of JAR–Ishikawa cell line co-culture was performed as follows. The Ishikawa cells were seeded at 1 × 10^5^ cells per well in a 24-well plate. The control group was untreated; LINC02190 group was transfected with LINC02190-overexpressing plasmids; LINC02190 + ITGAD group was transfected with LINC02190-overexpressing plasmids and ITGAD-overexpressing plasmids; siRNA-LINC02190 group was transfected with siRNA-LINC02190; siRNA-LINC02190 + anti-ITGAD group was transfected with siRNA-LINC02190 and incubated with 10 μg/mL anti-ITGAD antibody (abx101638, Abbexa) for 24 h. JAR cells were washed and adjusted to 5 × 10^6^/mL, stained with 1 μM Calcein-AM (Sigma) at 37°C, 5% CO_2_ in the dark for 30 min. Then diluted cell density to 1 × 10^6^/mL. The wells containing the Ishikawa cells were washed with PBS and refilled with RPMI 1640 medium containing 10% FBS. Then 100 μL JAR cells per well were added to each plate. The co-culture was maintained for 1 h at 37°C under 5% CO_2_ in the air. After incubation for 1 h, the culture medium was replaced with a fresh medium. The fluorescence-tagged cells were visualized and photographed by Zeiss LSM 510 confocal microscope (Zeiss), and Image J software (Image J 1.4, NIH, USA) was used for cell counting. Attachment rate was defined as the ratio of the number of attached cells to the total number of cells seeded. All experiments were replicated three times.

### Statistical analysis

Agilent Feature Extraction software (version 11.0.1.1) was used to analyze the acquired array images. Quantile normalization and subsequent data processing were performed using the GeneSpring GX v12.1 software package (Agilent Technologies). After quantile normalization of the raw data, LncRNAs and mRNAs at least five out of ten samples having flags in Present or Marginal (“All Targets Value”) were chosen for further data analysis. Differentially expressed LncRNAs and mRNAs with statistical significance between the two groups were identified through *P*-value/false discovery rate (FDR) filtering. Differentially expressed LncRNAs and mRNAs between the two samples were identified through fold change filtering. Pathway analysis and GO analysis were applied to determine the roles these differentially expressed mRNAs played in these biological pathways or GO terms. Hierarchical Clustering and combined analysis were performed using in-house scripts.

Statistical analysis was performed using the SPSS 23.0 software. All data were presented as mean ±s.d. A chi-square test was used for enumeration of the data and a *t-*test was used for quantitative data. The JAR cell attachment rate was compared between treatment groups using Fisher’s Exact test. *P* < 0.05 was considered statistically significant.

## Results

### Different lncRNA and mRNA expression of endometrial tissues in RIF patients

**Figure 1:**
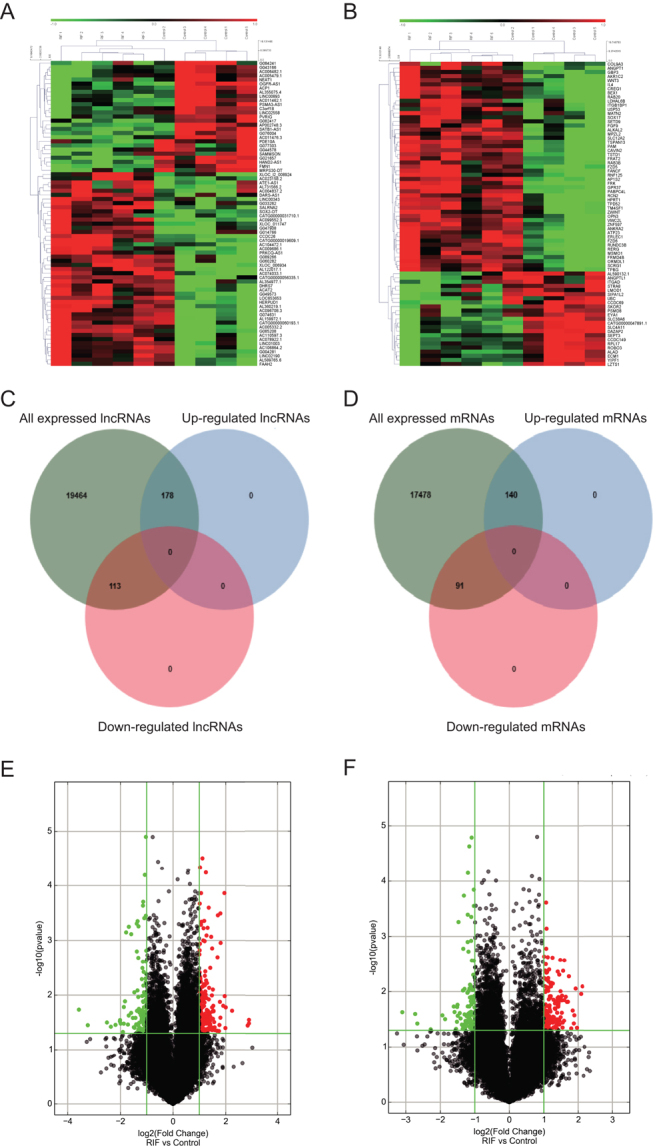
Different lncRNA and mRNA expression of endometrial tissues in RIF patients compared with control patients. Heat map, Venn diagram, and volcano plot of the differential lncRNA (A, C, and E) and mRNA (B, D, and F) expression analysis.

A total of 291 lncRNA transcripts were significantly differentially expressed in RIF endometrial samples compared with that in the control samples, with 178 lncRNA transcripts identified to be consistently up-regulated and 113 lncRNA transcripts downregulated (FC ≥ 2 and *P* < 0.05). A heat map, Venn diagram, and volcano plot of the differential lncRNA in endometrial tissues of RIF patients are shown in Fig. 1A, C and E. NR_120495, ENST00000618971, MICT00000222972, ENST00000522494, and ENST00000553986 were the most upregulated lncRNA transcripts. In comparison, ENST00000527092, ENST00000431361, ENST00000484125, T357032, and T330024 were the top downregulated lncRNA transcripts.

A total of 233 mRNA transcripts were significantly differentially expressed in endometrial samples of the RIF group compared with the control group. 142 RNA were significantly upregulated, and 91 were downregulated. A heat map, Venn diagram, and volcano plot of the differential mRNA in endal tissues of RIF patients are shown in Fig. 1B, D and F. ENST00000303921, ENST00000606080, ENST00000320963, ENST00000517746, and ENST00000278937 were the most upregulated mRNA transcripts. ENST00000354886, HBMT00000799578, ENST00000396426, ENST00000380059, and ENST00000549555 were the most downregulated mRNA transcripts. Table 2 listed the top 20 upregulated and downregulated lncRNA transcripts in our microarray data.

**Table 2:** Top 20 upregulated and downregulated lncRNA transcripts in RIF endometrial tissues.

Upregulated lncRNAs			Downregulated lncRNASs		
Probe name	GeneID	Fold change	Probe name	GeneID	Fold change
ASHGV40006307V5	ATE1-AS1	7.5957819	ASHGV40007205V5	ENSG00000255517	11.9012736
ASHG19LNC1A100048607V5	ENSG00000276742	7.5882537	ASHGV40004143V5	ENSG00000229873	9.427735
ASHG19LNC1A103690563V5	CATG00000056335	7.3468677	ASHG19LNC1A100100719V5	ENSG00000143727	5.6901268
ASHG19LNC1A100015738V5	ENSG00000254205	4.7336282	ASHGV40051636V5	G084241	5.1308904
ASHG19LNC1A101339951V5	ENSG00000100612	4.0051429	ASHGV40046878V5	G077303	4.5385191
ASHGV40020651V5	LOC653653	3.9654513	ASHGV40044376V5	ENSG00000112541	4.0470954
ASHG19LNC1A104431971V5	ENSG00000242808	3.8946981	ASHGV40026633V5	G043166	4.0288741
ASHGV40004847V5	ENSG00000229140	3.8645759	ASHGV40001061V5	ENSG00000228675	3.9653393
ASHG19LNC1A100068455V5	ENSG00000257241	3.7434796	ASHG19LNC1A100044409V5	ENSG00000259005	3.7409856
ASHG19LNC1A100032486V5	ENSG00000267731	3.5025331	ASHG19LNC1A109193468V5	ENSG00000241769	3.6564772
ASHGV40055173V5	G089266	3.4808758	ASHGV40051066V5	G082417	3.6504096
ASHG19LNC1A100051630V5	ENSG00000228521	3.478605	ASHG19LNC1A104008563V5	ENSG00000228956	3.5373692
ASHG19LNC1A100036657V5	ENSG00000273584	3.4340188	ASHG19LNC1A110860318V5	ENSG00000237125	3.4356961
ASHGV40046165V5	G074631	3.3917928	ASHG19LNC1A109848777V5	ENSG00000255730	3.276089
ASHGV40012607V5	G004281	3.3548754	ASHG19LNC1A100080830V5	ENSG00000259001	3.2089813
ASHGV40053005V5	G085208	3.2538541	ASHGV40027047V5	G044578	3.0093412
ASHG19LNC1A100063740V5	ENSG00000226669	3.184257	ASHGV40012484V5	G021657	3.0040735
ASHGV40048701V5	ENSG00000261455	3.0050985	ASHG19LNC1A114603925V5	ENSG00000088543	2.8762937
ASHG19LNC1A100003872V5	ENSG00000269489	2.9884548	ASHGV40056116V5	ENSG00000257621	2.8064567
ASHG19LNC1A101814936V5	ENSG00000120437	2.9515432	ASHGV40046567V5	G076004	2.766277

### Functional analysis of co-expressed mRNA

In the present study, we used GO and KEGG analysis to uncover the co-expressed mRNA of lncRNA to predict the functions of lncRNA. The GO analysis summarized the conceivable biological processes, cellular components, and molecular functions of the co-expressed mRNAs. The KEGG analysis summarized the potential pathways of the co-expressed mRNAs.

**Figure 2:**
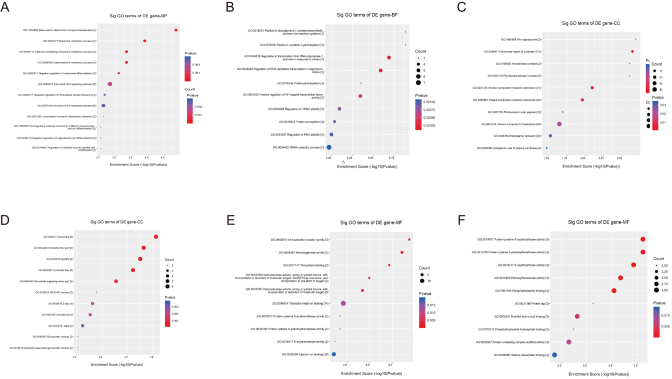
Top 10 GO terms of the significantly upregulated genes from the biological process (BP, A), molecular function (MF, C), and cellular component (CC, E) categories. Top 10 GO terms of the significantly downregulated genes from the biological process (BP, B), molecular function (MF, D), and cellular component (CC, F) categories.

Figure 2 showed the biological functions of the targets of differentially expressed lncRNA transcripts. The upregulated mRNAs mainly participate in biological processes such as beta-catenin destruction complex disassembly, dopamine metabolic process, and catechol-containing compound metabolic process (Fig. 2A). The downregulated mRNAs mainly participate in biological processes such as peptidyl-*S*-diacylglycerol-l-cysteine biosynthetic process from peptidyl-cysteine, peptidyl-l-cysteine S-palmitoylation, and the regulation of transcription from RNA polymerase II promoter in response to stress (Fig. 2B). The upregulated mRNAs are mainly involved in synthesizing cellular components such as the Wnt signalosome, perinuclear region of cytoplasm, and amyloid-beta complex (Fig. 2C). The downregulated mRNAs are mainly involved in synthesizing cellular components such as the sarcomere, contractile fiber part, and myofibril (Fig. 2D). According to the GO molecular function analysis, the upregulated mRNAs play important roles in Wnt-activated receptor activity, monooxygenase activity, and Wnt-protein binding (Fig. 2E). The downregulated mRNAs play important roles in protein-cysteine S-acyltransferase activity, protein-cysteine *S*-palmitoyltransferase activity and *S*-acyltransferase activity (Fig. 2F).

**Figure 3:**
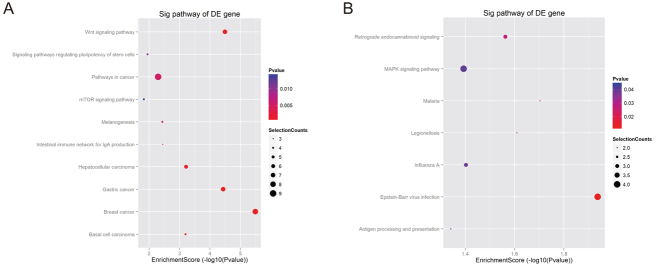
KEGG pathway enrichment analysis of the significant upregulated (A) and downregulated (B) genes.

The KEGG analysis revealed that the upregulated targets significantly participate in the following pathways: breast cancer, Wnt signaling pathway, and gastric cancer (Fig. 3A). The downregulated genes significantly participate in epstein–barr virus infection, malaria, and legionellosis pathways (Fig. 3B).

### Expression of LINC02190 and integrin αD (ITGAD) in the endometrium of RIF and control group

**Figure 4:**
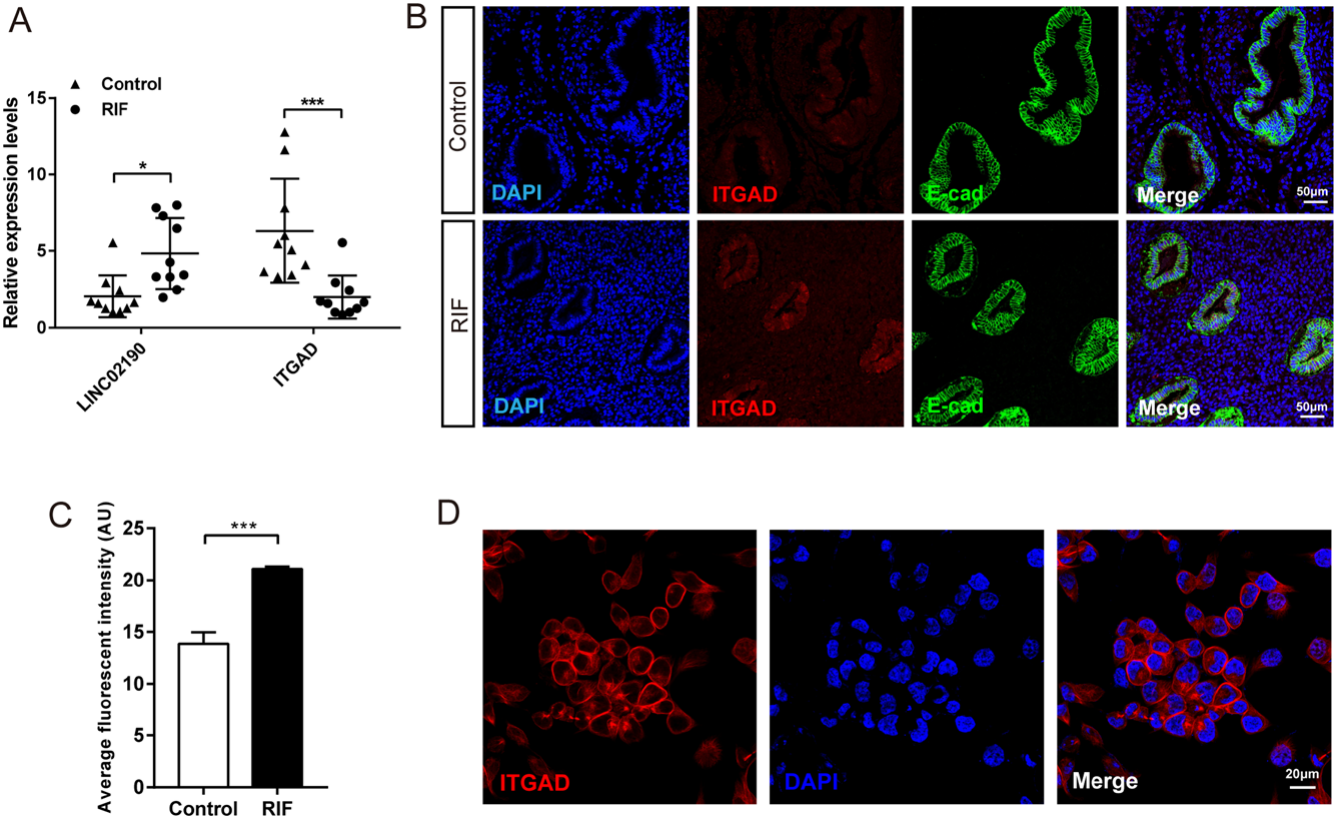
Expression and localization of LINC02190 and ITGAD in the endometrium of RIF and control group. LINC02190 and ITGAD relative expression level were verified in the endometrium samples from the RIF group and the control group by qPCR (A). Immunofluorescence assays of ITGAD in endometrial tissue from RIF patients and normal women. Nuclei were DAPI stained. ITGAD, red; E-Cadherin, green; cell nuclei, blue. Scale bar = 50μm. (B) Average fluorescent intensity of ITGAD in endometrial tissue from RIF patients and normal women was quantified by detecting fluorescent signal using the software Image J (C). Immunofluorescence assays of ITGAD in Ishikawa cell line. Scale bar = 20μm (D).

Having identified the potential functions of differentially expressed lncRNA, we further focused on the lincRNAs which had diverse features and functions such as remodeling chromatin and genome architecture, RNA stabilization, and transcription regulation, including enhancer-associated activity. Relationship for differential expression of lincRNAs (the lincRNA reference catalog was reported by Cabili *et al.* (2011)) between the RIF group and the control group (FC ≥ 2 and *P* < 0.05) and their adjacent mRNAs (distance < 200 kb) were predicted by using association analysis (Table 3). Since we sought to define the specific lncRNA that may regulate embryo implantation, ITGAD which is involved in cell–matrix adhesion and integrin-mediated signaling pathway was identified (Finalet Ferreiro *et al.* 2014). Among those, LINC02190 was markedly and highly expressed in the RIF group, and the results of association analysis suggested that downstream ITGAD mRNA expression may be regulated by LINC02190. To verify the results of LncRNA Microarray analysis, ten samples from the RIF group and control group were used to evaluate the expression level of LINC02190 and ITGAD by qRT-PCR. The results showed that LINC02190 was upregulated in the RIF group, while ITGAD mRNA was downregulated, which was consistent with the LncRNA microarray analysis results (Fig. 4A). To validate the expression level and localization of ITGAD, we performed immunofluorescence in the endometrium of patients in the RIF/control group. We found that ITGAD was mainly located in the glandular and luminal epithelia in endometrial tissues, which was co-located with E-cadherin, a widely-used epithelial marker (Fig. 4B). The expression of ITGAD was significantly decreased in the RIF group (Fig. 4B and C), in accordance with the result of mRNA detection. For the feasibility of the following experiments, ITGAD was also verified to be expressed mostly in the cell membrane of Ishikawa cells (Fig. 4D). Collectively, we demonstrated that LINC02190 was upregulated and ITGAD showed decreased expression in the endometrium of RIF compared to the control group.

**Table 3:** Association analysis of differential expressed lincRNAs between RIF group and the control group and their adjacent mRNAs.

LncRNAs	*P*-value	Fold change	Regulation of LncRNAs	Genome Relationship	Nearby mRNA	Regulation of mRNAs
ACP1	0.031564537	5.6901268	Down	Downstream	ALKAL2	Up
LOC653653	0.039883269	3.9654513	Up	Upstream	USP32	Down
HAND2-AS1	0.000714461	3.4356961	Down	Upstream	SCRG1	Up
AC011462.1	0.034411813	3.276089	Down	Upstream	HNRNPUL1	Down
LINC02190	0.033438155	2.5544443	Up	Downstream	ITGAD	Down
LINC01934	0.048464904	2.3554241	Up	Downstream	ITGA4	Up
AC074386.1	0.005143792	2.2730345D	Down	Downstream	ARHGEF35	Up
AC079779.1	0.033583922	2.2718636	Up	Downstream	ALKAL2	Up
BARD1	0.030244714	2.2083819	Up	Downstream	VWC2L	Up
PDE11A	3.08493E-05	2.1700167	Up	Downstream	TTC30B	Up
EYA1	0.040633487	2.160738	Down	Downstream	EYA1	Down
CATG00000057705.1	0.044766356	2.0900085D	Down	Upstream	NPTXR	Up
PPP1R10	0.0003563	2.0435753	Down	Upstream	IER3	Down

### LINC02190 could directly bind to ITGAD mRNA in Ishikawa cells

**Figure 5:**
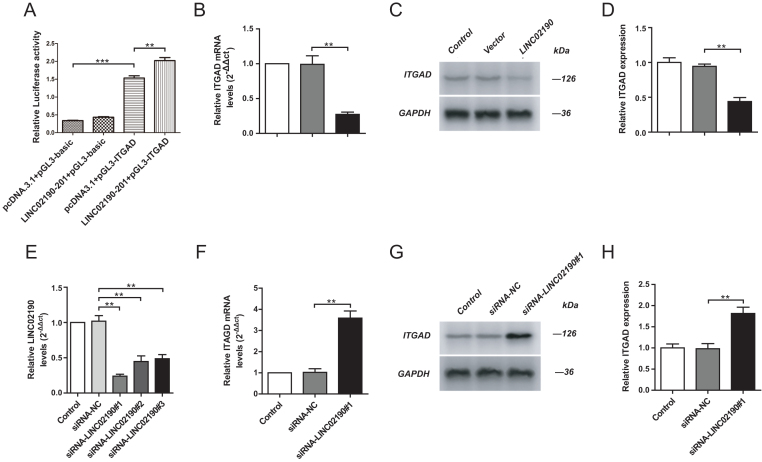
The effects of LINC02190 on ITGAD level in Ishikawa cells. LINC02190 was stably overexpressed in Ishikawa cells. The luciferase reporter assay of LINC02190 and ITGAD (A). The mRNA levels of ITGAD in the upregulated group was measured by qPCR (B). The ITGAD expression in the upregulated group was detected by western blot assay (C and D). The Ishikawa cells were stably knockdown LINC02190. The transfected efficiency detected by qRT-PCR, siRNA-LINC02190 #1 exhibiting the highest transfection efficiency was selected by qPCR for silencing expression (E). The mRNA levels of ITGAD in the downregulated group was measured by qPCR (F). The ITGAD expression in the downregulated group was detected by western blot assay (G and H). Results were mean ± s.d. for three individual experiments which, for each condition, were performed in triplicate. *P < 0.05, **P < 0.01, ***P < 0.001.

To identify whether LINC02190 directly regulated ITGAD mRNA expression, the dual-luciferase reporter assay for target validation was performed. Ishikawa cells transfected with pcDNA3.1-LINC02190-201+pGL3-ITGAD+PRL-TK showed significantly increased fluorescence intensity compared with Ishikawa cells transfected with pcDNA3.1+pGL3-ITGAD+PRL-TK, which indicated that LINC02190 could combine to ITGAD mRNA directly (Fig. 5A).

### Upregulated LINC02190 may reduce the expression of ITGAD

To explore the regulation of LINC02190 on ITGAD expression, we constructed LINC02190 overexpressed Ishikawa cell lines with the indicated plasmid. The transfected efficiency was determined by qRT-PCR. The results exhibited that LINC02190 expression was increased in Ishikawa cells treated with LINC02190 overexpressed plasmid.

After being transfected with LINC02190 overexpression plasmid or control plasmid (empty pcDNA3.1 vector), the expression of ITGAD mRNA in the Ishikawa cells was determined by qPCR, and the expression ITGAD was further checked by western blot. ITGAD mRNA and ITGAD protein were both significantly decreased in the LINC02190 group compared with the vector group (Fig. 5B, C, and D). Then, to knock down the expression level of LINC02190, the Ishikawa cells were transfected with siRNA-LINC02190 #1, siRNA-LINC02190 #2, and siRNA-LINC02190 #3, respectively. All the siRNA-treated groups showed decreased levels of LINC02190. However, the siRNA-LINC02190 #1 exhibiting the highest transfection efficiency was selected by qPCR for silencing expression in the following experiments (Fig. 5E). Moreover, after transfection with siRNA-LINC02190 #1 or siRNA-NC in the Ishikawa cells, ITGAD mRNA and ITGAD protein levels in siRNA-LINC02190 #1 group were significantly increased compared with the siRNA-NC group (Fig. 5F, G, and H), which indicated that the overexpression LINC02190 could reduce the expression of ITGAD to some extent.

### LINC02190 decreased the embryo–endometrial attachment by inhibiting ITGAD expression

**Figure 6:**
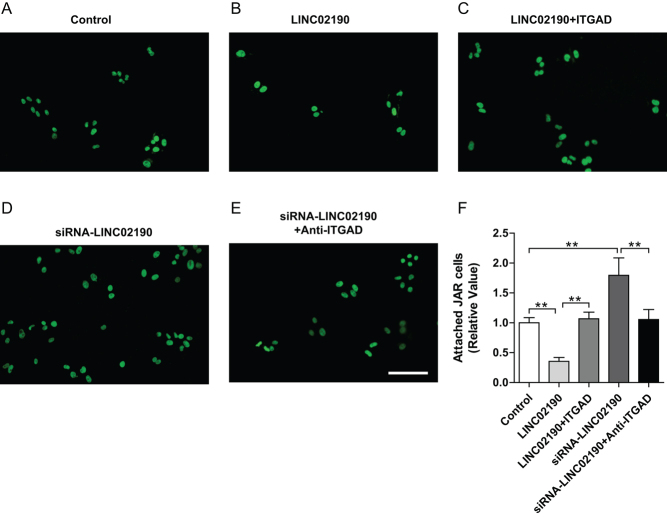
The effects of LINC02190 on embryo adhesion of Ishikawa cells. Ishikawa cells were untreated (A), and transfected with LINC02190-overexpressing plasmids (B), LINC02190-overexpressing plasmids + ITGAD-overexpressing plasmids (C), siRNA-LINC02190 (D) or siRNA-LINC02190 + anti-ITGAD antibody (E), respectively, and co-cultured with Calcein-AM stained JAR cells. The comparison result of adhesion rates in each group (F). Results were mean ± s.d. for three individual experiments which, for each condition, were performed in triplicate. *P < 0.05, **P < 0.01.

An *in vitro* human trophoblastic cell and endometrial cell co-culture model was used to investigate the effects of LINC02190 and ITGAD on embryo adhesion. The adhesion rate of Ishikawa and JAR cells was significantly reduced after the overexpression of LINC02190 in the Ishikawa cell (Fig. 6A and B). While, the inhibition of adhesion between Ishikawa cells and JAR cells was partially reversed after the co-overexpression of LINC02190 and ITGAD in Ishikawa cell (Fig. 6C), after knockdown of LINC02190, the adhesion rate of Ishikawa and JAR cells was increased obviously (Fig. 6D). However, the adhesion rate of JAR and LINC02190-knock down Ishikawa cells returned to normal after incubated with an anti-ITGAD antibody (Fig. 6E). Altogether, these results suggested that LINC02190 could decrease the embryo–endometrial attachment by inhibiting ITGAD expression.

### Identification of cis-elements involved in the regulation of ITGAD promoter activities

**Figure 7:**
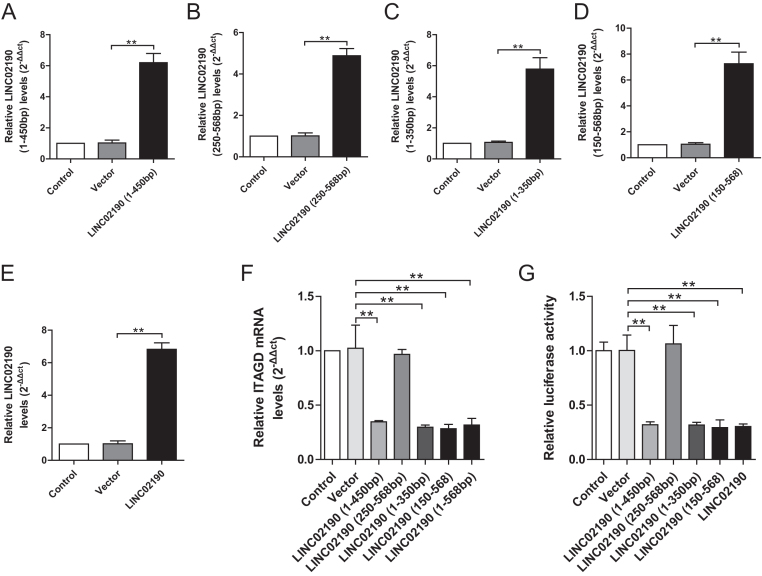
The transfected efficiency detected by qRT-PCR and the binding sites of LINC02190 and ITGAD were between 150 and 250 bp. The transfected efficiency of LINC02190 (1–450 bp, A), LINC02190 (250–568 bp, B), LINC02190 (1–350 bp, C), LINC02190 (150–568 bp, D), or LINC02190 (E) were stably overexpressed in Ishikawa cells. LINC02190 (1–450 bp), LINC02190 (250–568 bp), LINC02190 (1–350 bp), LINC02190 (150–568 bp), or LINC02190 was stably overexpressed in Ishikawa cells. And the mRNA levels of ITGAD (F) in the indicated group was measured by qPCR. The Luciferase assay (G). Results were mean ± s.d. for three individual experiments which, for each condition, were performed in triplicate. *P < 0.05, **P < 0.01.

LINC02190 (1–568 bp), LINC02190 (1–350 bp), LINC02190 (1–450 bp), LINC02190 (150–568 bp), and LINC02190 (250–568 bp) were overexpressed, respectively. The transfected efficiency of targeting different regions was determined by qRT-PCR. LINC02190 expression was all increased in Ishikawa cells treated with diverse LINC02190 overexpressed plasmid (Fig. 7A, B, C, D, and E). Luciferase reporter assay revealed the expression of ITGAD mRNA and the luciferase activity of ITGAD promotor in LINC02190 (1–450 bp) group, LINC02190 (1–350 bp) group, LINC02190 (150–568 bp) group, and LINC02190 group were significantly downregulated compared with the vector group, while the expression of ITGAD mRNA and the luciferase activity of ITGAD promotor in LINC02190 (250–568 bp) group were not significantly changed (Fig. 7F and G). This result suggested that the combined area of LINC02190 and ITGAD promotor was within the range of 150–250 bp.

## Discussion

Our study revealed the differentially expressed lncRNAs and mRNAs profiles in RIF compared with normal fertile female, and we further explored the possible mechanism of LINC02190 effecting on embryo–endometrial attachment. We found that LINC02190 could reduce ITGAD expression by binding to the 150–250 bp region of the ITGAD promoter, thereby impairing the embryo implantation, which may be one of the underlying molecular biological mechanisms of RIF.

About 1% of genes of the human genome were involved in protein-coding (Birney *et al.* 2007). LncRNAs, as a class of ncRNA, have been found to be involved in a series of biological processes, such as tumorigenesis (Bhan *et al.* 2017), development (Schmitz *et al.* 2016), and cardiovascular diseases (Lorenzen & Thum 2016), and most of them can be identified as the biomarkers of multiple diseases. RIF is a major obstacle of achieving clinical pregnancy in the process of ART, and it is also a hot topic and challenge in the field of reproductive medicine. Seeking new biomarkers and elucidating the possible mechanism of RIF from molecular biology not only help clinicians and researchers to have a deeper understanding of RIF but also provide potential biomarkers and targets for the intervention of RIF. In our study, Arraystar Human LncRNA Microarray was performed to identify the lncRNA and mRNA profiles of RIF patients. A total of 301 unique different expression lncRNAs were identified (181 lncRNAs were significantly upregulated and 120 were downregulated), and 233 unique mRNAs were identified (142 mRNAs were significantly up-regulated, and 91 were down-regulated). These identified lncRNAs might be used as potential biomarkers for the occurrence of RIF.

As one of the influencing factors of RIF, lncRNAs in the endometrium, oocytes, and cumulus cells are widely involved in immunocompetence, cell adhesion, apoptosis, chromatin remodeling to affect endometrial receptivity, embryo quality, and the synchronous development of the embryo and endometrium. LncRNA AK124742 is an antisense lncRNA of PSMD6*.*LncRNA AK124742 and PSMD6 rise synchronously around the cumulus cells of oocytes that develop into high-quality embryos (Li *et al.* 2015). LncRNAs (BCAR4, C3orf56, TUNAR, OOEP-AS1, CASC18, and LINC01118) which are significantly expressed in MII oocytes may be involved in chromatin remodeling, cellular pluripotency, and early embryonic development promotion (Bouckenheimer *et al.* 2018). LncRNAs (NEAT1, MALAT1, ANXA2P2, MEG3, IL6STP1, and VIM-AS1) which are significantly expressed in cumulus cells may be related to apoptosis and extracellular matrix-related gene expression (Bouckenheimer *et al.* 2018). Zeng *et al*. found that the expression of lncRNA H19 and ITGB3 in endometrial tissues of RIF patients were both decreased and positively correlated, which might be attributed to the effect that lncRNA H19 regulated ITGB3 through decreasing the expression of let-7 (Zeng *et al.* 2017). A recent study reported that lncRNA ENST00000433673 might upregulate the ITGAL mRNA by promoting the expression of ICAM1 mRNA and the adhesion of EECs, which facilitates the embryo–endometrial attachment (Li *et al.* 2019). Taken together, these studies indicated that lncRNAs played an important role in the occurrence of RIF.

For embryo implantation, endometrial receptivity is an important factor in the ability of the endometrium to allow embryo attachment (Lessey & Young 2019). HOXA10 (Chen *et al.* 2016), mucins (Chen *et al.* 2016), and leukemia inhibitory factor (Shokrzadeh *et al.* 2019) are important molecules in endometrial receptivity and implantation. Furthermore, the integrin family as cell surface receptors are widely involved in cell‐to‐cell and extracellular matrix adhesion (Qian *et al.* 2017). A variety of integrins could cause effects on early pregnancy by mediate invasion, cell-cell interaction, attachment, and decidualization, including α1β1, α3β1, α4β1, α4β3, αvβ3, αvβ1, αvβ5, and α6β1 (Rarani *et al.* 2018).

ITGAD, one of the α-subunits of the beta-2 integrin family, forms the leukocyte integrins αDβ2 by non-covalent association with β2-subunit (Azevedo-Quintanilha *et al.* 2019) and is involved in immunological synapse formation, cell-matrix adhesion, the proliferation of activated T-cells, and integrin-mediated signaling pathway (Finalet Ferreiro *et al.* 2014). Several studies showed that ITGAD could play a crucial role in cell adhesion. A microarray profiling in placenta tissues of preeclampsia and intrauterine growth restriction revealed that ITGAD was highly expressed and was associated with extracellular matrix organization (Medina-Bastidas *et al.* 2020). Hu *et al*. found that the methylation of ITGAD could be reduced by decidual natural killer cells conditioned medium, which was associated with differentiation, adhesion, and migration of extravillous cytotrophoblasts (Hu *et al.* 2012). In addition, ITGAD was also reported to be associated with the lymph node metastasis of non-small-cell lung cancer and the development of histiocytic sarcomas in dogs (Boerkamp *et al.* 2013, Li *et al.* 2020).

For the various functions of ITGAD in regulating biological progress, especially cell adhesion, we used the Ishikawa cell line, a well-differentiated human endometrial adenocarcinoma cell line and widely used as a model for investigation of endometrial function. JAR cell line, a choriocarcinoma cell line, has been used to research embryo implantation because of its invasion function and adhesiveness to Ishikawa (Hannan *et al.* 2010). An* in vitro* model using JAR and Ishikawa cell lines has been established to investigate embryo–endometrial attachment due to their proper characteristics. Several studies have reported to use this *in vitro* model in investigating embryo–endometrial attachment (Kim *et al.* 2016, Li *et al.* 2017, Yu *et al.* 2020, Cai *et al.* 2021). Due to the unavailability and ethical constraint of actual human blastocysts for experimental use, JAR–Ishikawa cell line co-culture may represent a reasonable surrogate, although the two may not be exactly identical. Thus, in this study, we used these *in vitro* co-culture models to evaluate the effect of LICN02190 and ITGAD expression levels on embryo-endometrial attachment.

A previous study demonstrated that activated ITGAD by p-STAT1 in coronary vascular tissues could exacerbate coronary microcirculation in atherosclerotic mouse model (Gao *et al.* 2021). ITGAD was also reported to have a low expression on neutrophils and monocytes in circulation, but upregulated on tissue macrophages, particularly in atherosclerotic lesions and adipose tissue during diabetes (Cui *et al.* 2019). High expression of ITGAD on the cell surface possesses a strong adhesion to extracellular matrix (ECM) proteins that leads to the retention of proinflammatory macrophages in inflamed tissue (Cui *et al.* 2019). Therefore, as it is involved in the adhesion potential, we inferred that decreased ITGAD in endometrium cells may impair the embryo–endometrial attachment due to the poor adhesion.

The integrin family is widely expressed on leukocytes and is essential to leukocyte interactions with cells and the ECM, which plays an important role in the inflammatory responses and vascularization (Sims & Dustin 2002). Inflammatory leukocytes that infiltrate the tissue premenstrually are considered to be a critical effector of menstruation (Hirota *et al.* 2005). These leukocytes in the endometrium can secrete a wide variety of inflammatory mediators, which would be capable of initiating the breakdown of the endometrium and promoting the proliferation of the endometrial cells, reepithelialization, and angiogenesis for the reconstruction of the shed endometrium (Hirota *et al.* 2005). Thus, it is conceivable that the high expression of LINC02190 in RIF may also decrease ITGAD in leukocytes and lead to poor endometrial remodeling due to disorder of inflammatory responses and vascularization.

In conclusion, our study found that there were multiple differentially expressed lncRNA and mRNA in endometrial tissue of RIF patients compared with normal fertile female, which could be used as potential biomarkers of RIF. Among them, LINC02190 highly expressed in the RIF group could decrease ITGAD expression by binding to the 150–250 bp region of the promoter and inhibit the embryo–endometrial attachment, which might be one of the underlying molecular biological mechanisms of RIF.

## Declaration of interest

The authors declare that there is no conflict of interest that could be perceived as prejudicing the impartiality of the research reported.

## Funding

This study was funded by Beijing Municipal Administration of Hospitals http://dx.doi.org/10.13039/501100009601 Clinical Medicine Development (grant numbers ZYLX201830) and Beijing Hospitals Authority’ Ascent Plan (grant numbers DFL20191401).

## Ethics approval and consent to participate

The study protocol was approved by the Ethics Committee of the Beijing Obstetrics and Gynecology Hospital, Capital Medical University.

## Availability of data and materials

All of our microarray gene expression information in this work was deposited in NCBI’s Gene Expression Omnibus and could be accessed through GEO series accession (GSE188409).

## Author contribution statement

Y X K proposed the study design. Z X H, W Q, L C, L Y, and L Y collected patients’ information. W S Y and L Y L checked the patients’ data and reviewed the calculations and statistical approaches. Z F Y and C T performed the experimental work, drafted the manuscript and constructed the tables under the guidance of Y X K and Y Y. All authors approved the final version of this article.
